# 167. Duration of Methicillin-Resistant *Staphylococcus aureus* (MRSA) Nares PCR Positivity while Receiving Highly Active Anti-Staphylococcal Drugs

**DOI:** 10.1093/ofid/ofad500.240

**Published:** 2023-11-27

**Authors:** Maximillian S Wu, Wesley D Kufel, Scott W Riddell, Jeff Steele, Elizabeth Asiago-Reddy

**Affiliations:** SUNY Upstate Medical University, Syracuse, New York; Binghamton University School of Pharmacy Sciences, Binghamton, New York; State University of New York Upstate Medical University, Syracuse, New York; Upstate University Hospital, Syracuse, New York; SUNY Upstate Medical University, Syracuse, New York

## Abstract

**Background:**

A growing body of evidence has demonstrated a close association between nasal colonization with methicillin-resistant *Staphylococcus aureus* (MRSA) and its invasive presence systemically. Thus, several hospitals have implemented MRSA nares PCR screening for all patients initiating treatment active against MRSA for various infections to help de-escalate an anti-MRSA antibiotic in the event of a negative result. However, little is known about the effects of anti-MRSA systemic agents on MRSA nasal colonization, potentially affecting the use of MRSA PCR as an effective screening tool if not conducted immediately upon hospital admission.

**Methods:**

Adult Upstate University Hospital patients ≥ 18 years of age with an initial positive MRSA nares PCR result were considered for inclusion, starting from September 2022. Eligible patients were individuals who had a positive MRSA nares PCR performed within 36 hours of their initial dose of a systemic anti-MRSA antibiotic. Positive MRSA nares cultures were also considered if PCRs were initially indeterminate. Anti-MRSA antibiotics included vancomycin, daptomycin, linezolid, ceftaroline, and clindamycin. Patients who were received these agents for a duration of 48-96 hours were consented for their participation. If consented, a follow-up MRSA nares swab and PCR were collected at a goal timepoint of 72 hours post initiation of anti-MRSA antibiotics (sample collection ranged from 48-96 hours post initiation). MRSA colonization was reassessed with a repeat nares PCR and reflex to culture for indeterminate results if applicable.

**Results:**

As of April 21^st^, 2023, 61 follow-up MRSA nares samples were collected from eligible participants. Follow-up samples were collected an average of 66.51 hours after their first dose of anti-MRSA drugs (IQR = 11.47). Of these follow-up samples, 56/61 (91.80%) resulted as a positive MRSA nares PCR or culture result.

**Conclusion:**

Our interim results demonstrated that MRSA colonization in the nares via PCR generally persists even after 48-96 hours of continuous systemic administration of anti-MRSA antibiotics. These findings suggest that MRSA nares swabs can still be used after anti-MRSA antibiotic administration, increasing its utility as a screening tool.

**Disclosures:**

**Wesley D. Kufel, Pharm.D., BCPS, BCIDP, AAHIVP**, Merck & Co.: Grant/Research Support **Elizabeth Asiago-Reddy, MD**, Glaxo-Smith-Kline: Grant/Research Support|Pfizer: Grant/Research Support

